# Neuronal Cell Types in the Spinal Trigeminal Nucleus of the Camel Brain

**DOI:** 10.3390/brainsci13020312

**Published:** 2023-02-12

**Authors:** Qasim A. El-Dwairi, Saleh M. Al-Hussain, Ayat S. Banihani, Ziad M. Bataineh, Laiche Djouhri, Ayman G. Mustafa, Sami Zaqout

**Affiliations:** 1 Department of Anatomy, Faculty of Medicine, Jordan University of Science & Technology, P.O. Box 3030, Irbid 22110, Jordan; 2 Department of Rehabilitation Sciences, Faculty of Allied Medical Sciences, Jordan University of Science & Technology, P.O. Box 3030, Irbid 22110, Jordan; 3 Department of Basic Medical Sciences, College of Medicine, QU Health, Qatar University, Doha P.O. Box 2713, Qatar

**Keywords:** camel, spinal trigeminal nucleus, neurons, Golgi

## Abstract

Neurons in the spinal trigeminal nucleus of a camel were morphologically studied by the Golgi impregnation method. The neurons were classified based on the size and shape of their cell bodies, the density of their dendritic trees, and the morphology and distribution of their appendages. At least 12 morphological types of neurons were found in the camel spinal trigeminal nucleus, including the following: stalked, islets, octopus-like, lobulated, boat-like, pyramidal, multipolar, round, oval, and elongated neurons. These neurons exhibited large numbers of various forms of appendages that arise not only from their dendrites but also from their cell bodies. Moreover, neurons with unique large dilatations especially at their dendritic branching points were also reported. The neurons reported in this study displayed an array of different sizes and shapes and featured various forms of appendages arising from cell bodies and dendrites. Such morphologically distinctive neuronal cell types might indicate an evolutionary adaptation to pain and temperature processing pathways at the level of the spinal trigeminal nucleus in camels, which traditionally live in a very harsh climatic environment and are frequently exposed to painful stimuli.

## 1. Introduction

The spinal trigeminal nucleus (STN) extends from the midpons to the upper three or four cervical spinal segments [1]. It is subdivided into three areas: the spinal trigeminal oralis (STO), the spinal trigeminal interpolaris (STI), and the spinal trigeminal caudalis (STC) [2,3].

Neurons in the STN have been studied in many species such as rats, cats, monkeys, and humans using different methods such as Golgi impregnation, horseradish peroxidase (HRP), Nissl stain, and immunocytochemistry. Several neuronal cell types were found in the three subdivisions of the STN. For instance, six types of neurons were found in the cat STC. These were either spiny pyramidal, aspiny pyramidal, multipolar with a dense dendritic tree, multipolar with sparsely branching dendrites, islet cells with small oval or round cell bodies and small clusters of spines on their distal dendrites, or stalked cells with numerous fine stalk-like branches and spines on their dendrites [4,5,6]. In a rat, one study [7] showed well-stained stalked cells. These stalked cells featured very extensive dendritic and axonal trees with a large number of stalks (thin side branches) arising from their dendrites. Other reports investigated the neuronal cell types in the human STC. Two main neuronal types were described [1,8]. The fist type was small rounded or fusiform cells (8–10 µm in diameter) which were either grouped in small clusters or dispersed. The second type was large neurons (22 µm in diameter) with a pear-shaped, fusiform, multipolar, or bipolar appearance.

The other two areas of the STN have also been studied extensively in different species. For example, five neuronal cell types were found in the cat STI [9]. These types were of various shapes including smooth pyramidal, smooth multipolar with spheroidal dendritic arborizations, bipolar fusiform or oval small somata, stalked neurons with 2–4 extensively ramified spiny dendrites, and islet cells with very small oval somata and dense dendrites. The sizes of the aforementioned neurons ranged from 6–12 µm to 15–25 µm in diameter. On the other hand, three main neuronal cell types were reported in a rat STO [10,11].

The shapes of the reported neuronal cell types were either oval, fusiform, or pyramidal in shape. The sizes of the neuronal somata ranged from 5–15 µm to 25–50 µm in diameter. Most of the previous studies classified neurons in the STN based on the shape and size of their cell bodies without giving much attention to the dendritic trees of the neuronal cells. The only exception was the detailed description of the stalked neurons in the STN of small animals such as the rat and cat [4,7,9]. These stalked neurons were described as medium neurons with large numbers of spiny stalks. Moreover, the dendritic trees were briefly described in two previous studies investigating the neuronal cell types in the human STC [1,8]. Interestingly, it has been shown that pain sensation in general is precieved by specialized sensory neurons with sophisticated dendritic processes to detect harmful stimuli [12,13].

The aim of this study is to describe the morphological features of different neuronal cell types in the camel STN and compare these neurons with their counterparts previously described in other species. The study will shed the light on the morphological features of the neuronal cell types in the camel STN that might be part of camels’ adaptation to processing pain and temperature sensations arising in the face.

## 2. Materials and Methods

The Golgi–Kopsch method of silver impregnation was used. This method has been extensively used and validated by our research group [14,15,16,17]. This method usually stains between 1 and 5% of neurons without overlapping between the stained neurons. This enabled us to study the different parts of neurons including their spines and appendages. Eight camel brains ranging in age from two to four years were collected from local butchery stores directly after sacrifice, kept for two to four hours in dd-H_2_O, and transferred to the lab where the brain stems were dissected and preserved in 10% formalin solution (CH_2_O; CP10.2, Carl Roth GmbH, Karlsruhe, Germany) for two to three months. No animals were sacrificed in this study; therefore, Institutional Animal Care and Use Committee (IACUC) approval was waived. Coronal blocks of brain stem between the inferior cerebellar peduncle and spinomedullary junction containing the spinal trigeminal nucleus were cut. The tissue blocks taken from the first 6 mm, 7–20 mm, and 21–30 mm below the inferior cerebellar peduncle represented the oral, interpolar, and caudal parts of the nucleus, respectively [9,18]. The blocks were then processed according to:

### 2.1. Fox et al. Modification [19]

A block (4 mm thick) containing the AV thalamic nucleus was cut, dried, and kept in a mixture of 3% zinc chromate (CrO_4_Zn; 11483697, Thermo Fisher Scientific Inc., Dreieich, Germany) and 2% formic acid (CH_2_O_2_; 15508664, Thermo Fisher Scientific Inc., Germany) for 7 days. Then, the block was removed from the chromate solution, dried without washing, and immersed in a 0.75% silver nitrate solution (AgNO_3_; 4500.1, Carl Roth GmbH, Germany) for another 7 days. This method gave the best results regarding the impregnation of axons of Golgi-type II neurons, with impregnation of very few neurons with a very clear background.

### 2.2. Braitenberg et al. Modification [20]

Blocks (about 2 mm thick) containing the anterior ventral nucleus of the thalamus were cut, dried, and placed in a mixture of 3% potassium dichromate (K_2_Cr_2_O_7_; 1.04862, Merck KGaA, Darmstadt, Germany), 0.5% formaldehyde (CH_2_O; CP10.2, Carl Roth GmbH, Karlsruhe, Germany), and 12.5% sucrose (C_12_H_22_O_11_; 1.07687, MerckKGaA, Darmstadt, Germany) for 12 days. Then, they were incubated in a 0.75% silver nitrate solution (AgNO_3_; 4500.1, Carl Roth GmbH, Germany) for 7 days.

These two modifications were tested at the beginning of the experimental setup in order to select the one with the best impregnation outcome at the area of the STN. The neurons described in this study were impregnated using Braitenberg et al. modification [20].

### 2.3. Sectioning and Mounting

The blocks of the two modifications were removed from the silver nitrate and then cut into 100 μm-thick sections using a sliding microtome. The sections were kept in absolute alcohol (C_2_H_6_O; K928.4, Carl Roth GmbH, Germany) in their original serial order for 2 h before being transferred to xylene (C_8_H_10_; 9713.3, Carl Roth GmbH, Germany) for clearing where they were kept for 5 min. The sections were mounted on slides using DPX mounting medium (100579, Merck KGaA, Germany). Lastly, the slides were covered with cover slips.

### 2.4. Drawings and Photographs

The area located between the reticular formation and the lateral border of the medulla oblongata defined the estimate boundaries of the spinal trigeminal nucleus [9]. The neurons of reticular formation were distinguished based on their mega-size character. Selected well-impregnated neurons were studied under a Nikon light microscope equipped with an oculometer and imaging system. A neurite tracer plug-in in Fiji/ImageJ was used to draw representative neurons.

## 3. Results

To study the neurons of the sub-nuclei of the STN, blocks of the brainstem at the level of the STC, STI, and STO were impregnated using the Golgi technique. The neurons in the STN were classified according to:

1. Soma size (small: mean diameter = 15 µm or less, medium: mean diameter 16–24 µm, large: mean diameter 25–35 µm, and very large: mean diameter more than 35 µm).
2. Soma shape (round, oval, elongated, pyramidal, triangular, octopus-like, boat-like, multipolar, or lobulated.
3. Density of dendritic trees.
4. The presence of different types of appendages (protrusions, spines, grape-like appendages, and thin side branches).
5. Density and distribution of appendages on dendrites and/or cell bodies and or axons.

Similar neuronal types were found in the three subdivisions of the STN. Therefore, these neuronal types will be described together. Based on the criteria used in this study, at least twelve types (type I–type XII) of neurons were found in each subdivision (STC, STI, and STO) of the STN. 

### 3.1. Type I (Stalked) Neurons

**Figure 1:**
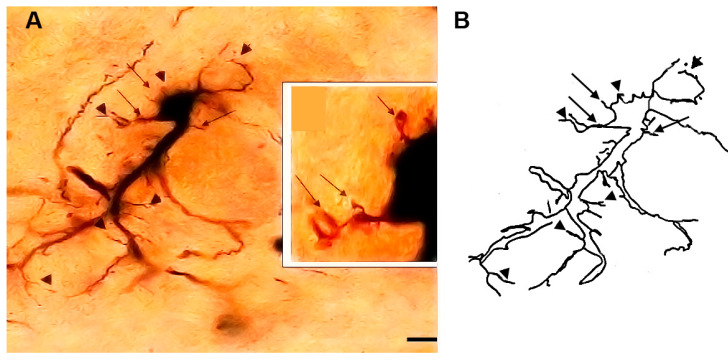
Photomicrograph (**A**) and drawing (**B**) of a type I neuron (stalked cell). Black arrows indicate somatic appendages while the arrowheads indicate dendritic appendages. The inset in (**A**) shows a magnified view of the soma. Scale bar = 15 µm.

**Figure 2:**
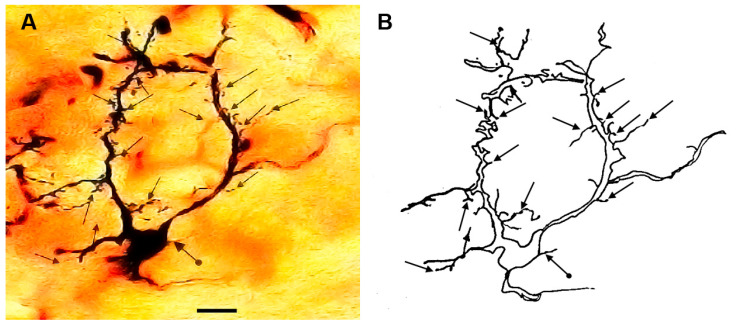
Photomicrograph (**A**) and drawing (**B**) of another type I stalked cell. Arrows indicate different form of dendritic appendages while dot arrows indicate somatic side branches. scale bar = 14 µm.

These neurons (Figure 1 and Figure 2) represented the most common neuronal type impregnated in the three subdivisions of the STN. Their somata diameters ranged from 13 to 15 µm (mean diameter = 14 µm ± 0.3, *n* = 40). Their somata exhibited round, oval, or multipolar shapes. Different types of appendages (somatic spines, grape-like appendages, and thin side branches) were commonly seen on the cell bodies in the neurons of this type (Figure 1 and Figure 2). Few primary dendrites [2,3,4,5] emerged from the cell body. These dendrites showed moderate arborizations. The most characteristic feature of these neurons is the large number of spines, grape-like appendages, and spiny thin side branches of different lengths and shapes (Figure 1 and Figure 2). Therefore, these cells can be described as stalked cells due to their large number of side branches that look like stalks. The axons of these cells were not impregnated in this study.

### 3.2. Type II (Islet) Neurons

**Figure 3:**
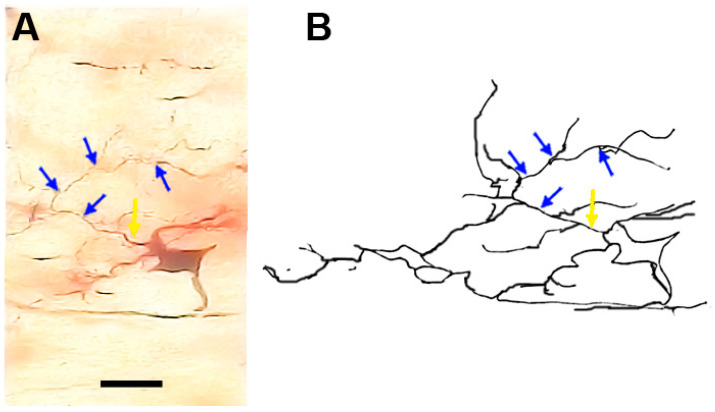
Photomicrograph (**A**) and drawing (**B**) of a type II Islet cell with a branched local axon. The arrows point to the axon and its branches. Scale bar = 15 µm.

The somata of these small neurons exhibited round, oval, or elongated shapes. The somatic mean diameter was less than 15 µm and each soma gave rise to 1–3 dendrites. These dendrites were either short or moderate in length, with a variable number of spines or with no spines at all. Moreover, the dendrites were either unbranched or had few branches. Axons with several branches of these neurons were impregnated in this study. Based on differences regarding their sizes, the neurons of this group were classified into two subtypes (Type IIa and Type IIb). (Figure 3).

### 3.3. Type IIa

These neurons (Figure 3) were small with somata diameters ranging from 6 to 10 µm (mean diameter = 7.5 µm ± 0.5, *n* = 20). Their somata exhibited round, oval, or elongated shapes. Each neuronal soma gave rise to 1 to 3 dendrites. These dendrites were either short or moderate in length, with a variable number of spines or without any spine at all. They were either unbranched or had few branches.

### 3.4. Type IIb

These neurons (not shown) were small with somata mean diameters ranging from 11 to 15 µm (mean diameter = 13 µm ± 0.6, *n* = 15). Their somata primarily exhibited an elongated or fusiform shape. They had 2–3 dendrites. These dendrites were either short or moderate in length with no spines or with a different number of spines. Moreover, they were unbranched or had few branches.

### 3.5. Type III Neurons

**Figure 4:**
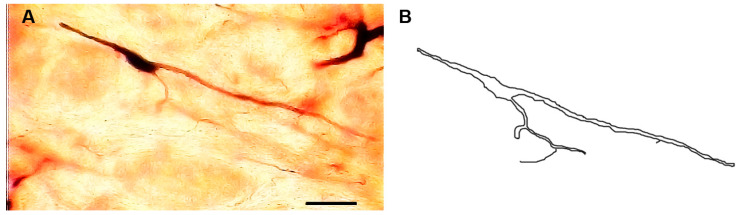
Photomicrograph (**A**) and drawing (**B**) showing type III neurons with generally smooth dendrites. Scale bar = 25 µm.

These neurons (Figure 4) were medium-sized neurons. Their somata exhibited fusiform or elongated shapes with somata diameters ranging from 16 to 25 µm (mean diameter = 20 µm ± 2.1, *n* = 10). Two to four dendrites emerged from the cell body. They were either unbranched or had few branches. Moreover, these dendrites were generally smooth but some of them had spines or protrusions. 

### 3.6. Type IV Neurons

**Figure 5:**
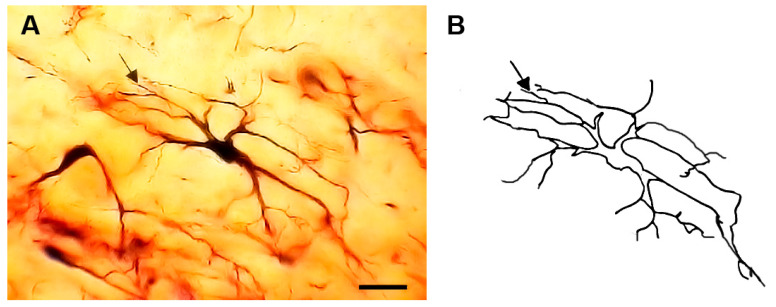
Photomicrograph (**A**) and drawing (**B**) of a type IV neuron with terminal side branches indicated by a black arrow. Scale bar = 30 µm.

These neurons (Figure 5) were large with somata diameters ranging from 26 to 35 µm (mean diameter = 31 µm ± 2.2, *n* = 12). Their somata exhibited multipolar, fusiform, or elongated shapes. Between 4–6 dendrites emerged from the cell body. These dendrites had a moderate number of branches and some of them gave several side branches at their ends. 

### 3.7. Type V Neurons

**Figure 6:**
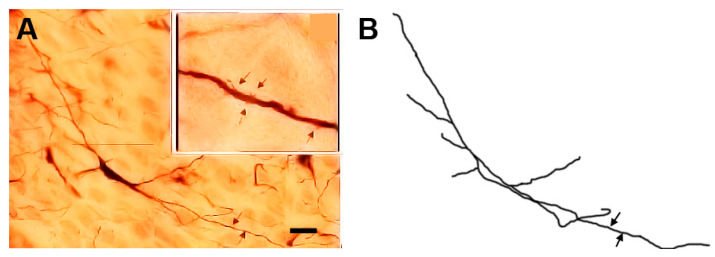
Photomicrograph (**A**) and drawing (**B**) showing a type V neuron. Arrows indicate dendritic protrusions, spines, and appendages. The inset in (**A**) shows a magnified view of one of the dendrites. Scale bar = 80 µm.

These neurons (Figure 6) were very large with somata diameters greater than 35 µm (mean diameter = 55 µm ± 4.2, *n* = 8). Their somata exhibited an elongated or fusiform shape. They gave rise to 4–6 dendrites with few branches and a moderate number of protrusions, spines, and appendages.

### 3.8. Type VI (Pyramidal) Neurons

**Figure 7:**
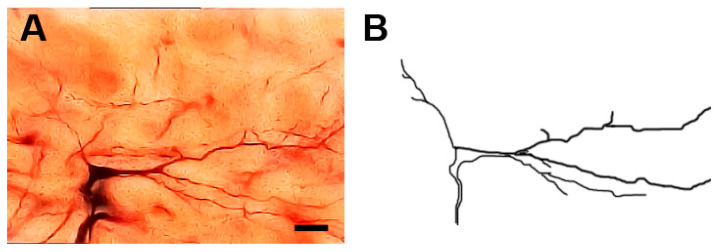
Photomicrograph (**A**) and drawing (**B**) of a type VI smooth pyramidal neuron. Scale bar = 20 µm.

These neurons were pyramidal in shape with somata diameters ranging from 14 to 20 µm (mean diameter = 16 µm ± 1.2, *n* = 12). They possessed one apical dendrite and two basal dendrites. The apical dendrite was long with a moderate number of branches while the basal dendrites were short with a lower number of branches. These neurons were generally smooth (Figure 7). 

### 3.9. Type VII (Triangular) Neurons

**Figure 8:**
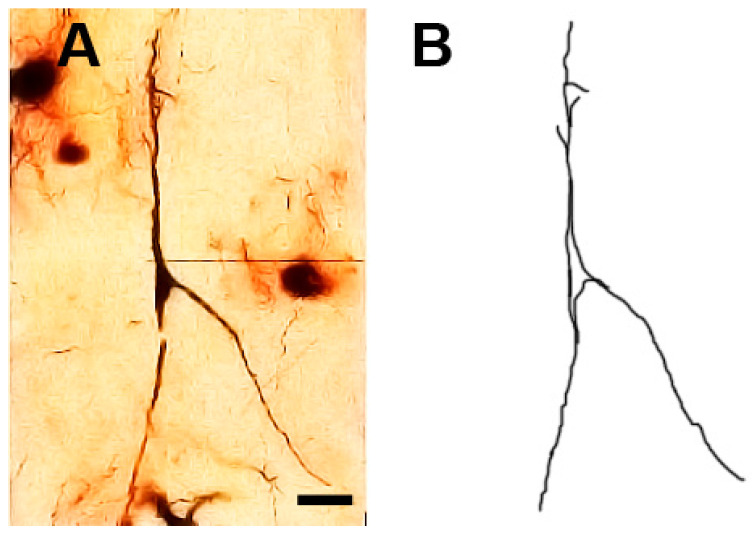
Photomicrograph (**A**) and drawing (**B**) of a type VII triangular neuron with three smooth dendrites. Scale bar = 40 µm.

These neurons (Figure 8) had triangular-shaped somata with diameters ranging from 26 to 35 µm (mean diameter = 30 µm ± 1.8, *n* = 10). Three dendrites emerged from the cell body in different directions. The dendrites were primarily smooth with no spines or with some protrusions. They were either unbranched or had very few branches.

### 3.10. Type VIII (Octopus-like) Neurons

**Figure 9:**
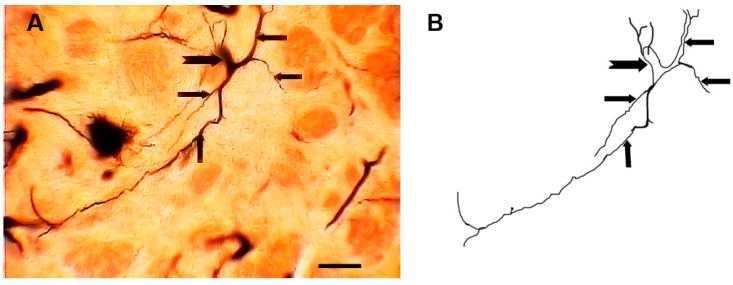
Photomicrograph (**A**) and drawing (**B**) of a type VIII octopus-shaped neuron with long, generally smooth dendrites (arrows). The notched arrows indicates the cell body. Scale bar = 25 µm.

These neurons (Figure 9) displayed octopus-like somata with diameters ranging from 15 to 18 µm (mean diameter = 17 µm ± 0.6, *n* = 16). The cell bodies displayed some spines and appendages and gave rise to 2–3 dendrites. The dendrites were either short or long with different types of appendages. Some of the dendrites were without spines. Regarding branching, the dendrites either had a few branches or were unbranched. 

### 3.11. Type IX (Boat-like) Neurons

**Figure 10:**
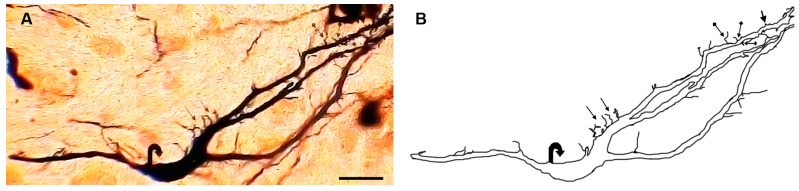
Photomicrograph (**A**) and drawing (**B**) of a type IX boat-like neuron with long dendrites that have large numbers of thick stalks, side branches (black arrows), and other types of appendages (black dot arrows). The cell body is indicated by U-turn arrows. Scale bar = 60 µm.

These neurons (Figure 10) displayed boat-like somata. The somata were generally smooth with diameters ranging from 20 to 40 µm (mean diameter = 30 µm ± 3.8, *n* = 8). Two to three dendrites emerged from each cell body. These dendrites were either short or long and with a highly variable number of branches. In addition to this, the dendrites displayed variable numbers of thick stalks, appendages, and spines. However, some dendrites were without spines.

### 3.12. Type X (Neurons with Swellings) Neurons

**Figure 11:**
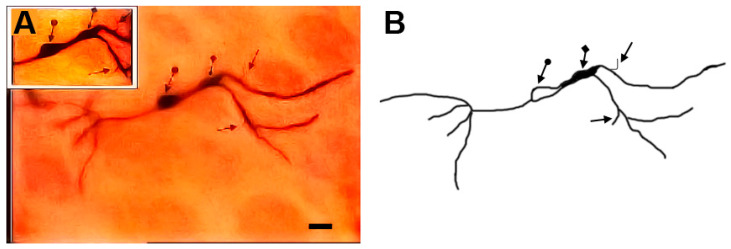
Photomicrograph (**A**) and drawing (**B**) of a type X neuron (a neuron with swelling). The dot arrows indicate the cell body. The diamond arrows indicate swelling at the dendritic branching point and the black arrows indicate to the dendritic side branches. The inset in (**A**) shows a focused view of the soma. Scale bar = 20 µm.

**Figure 12:**
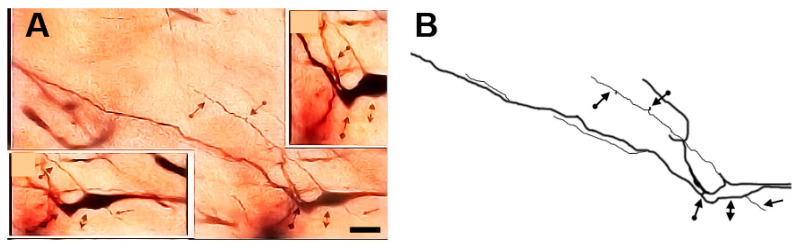
Photomicrograph (**A**) and drawing (**B**) of a type X neuron with swelling. In the figure and insets (**A**,**B**), the bidirectional arrows indicate the cell body while the diamond arrows indicate one swelling. The arrows indicate a somatic side branch while the dot arrows indicate dendritic protrusions. Scale bar = 30 µm.

These neurons (Figure 11 and Figure 12) were characterized by having dilated parts especially at the branching points of their dendrites. In some cases, these dilated parts had a diameter equal (or close to) the diameter of their cell bodies. Moreover, the neuronal somata were highly variable in size and shape. These neurons were generally smooth but some of them had small numbers of protrusions, spines, and side branches arising from their cell bodies and/or dendrites.

### 3.13. Type XI (Lobulated) Neurons

**Figure 13:**
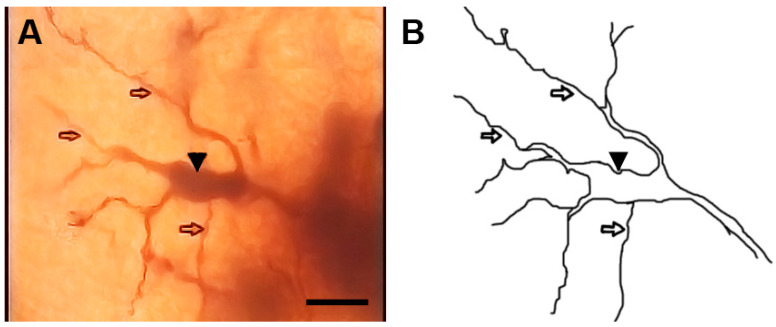
Photomicrograph (**A**) and drawing (**B**) of a type XI lobulated neuron with beaded dendrites (arrows). The arrowhead indicates the cell body. Scale bar = 30 µm.

These neurons (Figure 13) were lobulated with somata diameters ranging from 16 to 24 µm (mean diameter = 17 µm ± 1.3, *n* = 8). The neuronal somata had 4 to 6 dendrites with a variable number of appendages. Some dendrites of these neurons were beaded but they lacked any spines.

### 3.14. Type XII (Multipolar) Neurons

**Figure 14:**
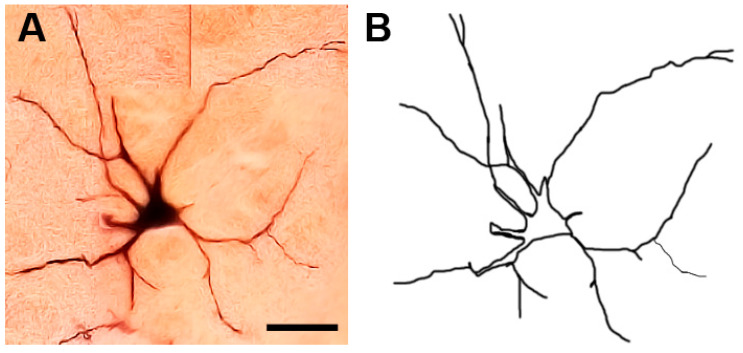
Photomicrograph (**A**) and drawing (**B**) of a type XII multipolar neuron with smooth dendrites. Scale bar = 30 µm.

These neurons (Figure 14) displayed multipolar somata with diameters ranging from 15 to 30 µm (mean diameter = 22 µm ± 3.2, *n* = 6). They had 4 to 7 primary dendrites emerging from the cell body. These dendrites gave rise to several branches with generally smooth surfaces. 

## 4. Discussion

Camels live in a unique environment with harsh climatic conditions and they eat thorny plants. They are exposed to painful stimuli (dust and thorny plants) and extreme temperatures. These sensations in the face area may affect the development of neurons in the camel’s STN, which houses the second-order neurons of the sensory pathways for pain and temperature for the face area. Therefore, characteristic and unique morphological neuronal features in the camel STN might be part of the adaptation process to the harsh climatic conditions of the camels’ habitat.

Neurons in the STN of rats, cats, and humans have been studied in the past using different techniques such as Nissl stain, Golgi impregnation, horse-radish peroxidase (HRP), and immunocytochemistry [1,4,5,6,7,8,9,10,11,21,22]. Only the stalked neurons (type I neuron in this study) were described in detail [4,7,9]. These neurons were described as complex neurons with spinous dendrites and a large number of spiny dendritic stalks. Interestingly, spine dynamics are mostly affected by neuronal activity and developmental age [23]. Other neurons (islet, fusiform, pyramidal, and multipolar) have been described in previous studies without much detail. Both spiny and aspiny pyramidal neurons were described in a cat [5]. The islet cells were described as small, round, or oval cells with some spines on their dendrites [4]. Other morphological details of these cells such as the density of dendritic trees and the types and distribution of different forms of appendages remain unreported. To the best of our knowledge, this is the first study to investigate the morphology of the neurons in the camel STN. In summary, the findings of this study confirm the complex nature of the stalked neurons in the STN. Moreover, it provides a detailed description of other neurons such as the islet, fusiform, pyramidal, and multipolar, which have only been briefly described in previous reports. Our study reports for the first time neurons with characteristic features, such as octopus-like, boat-like, and lobulated cells. In addition to this, the study reports cells with unique dilatations especially at the branching points of their dendrites. 

This study reports at least twelve types of nerve cells in the camel STN. They were similar in all three components of the STN, namely the STC, STI, and STO. These neuronal cell types in the three components of the camel STN were compared with the neuronal cell types described in previous Golgi studies in the STN of other species namely a rat, a cat, and a human. 

Most previous morphological studies of the neurons in the STN in particular species (a rat, a cat, and a human) have classified neuronal cells in this sensory nucleus according to the soma size and shape using different techniques. The description of dendritic trees and somatic and/or dendritic spines and appendages was insufficient. The only exception was the stalked cells, which have been described previously in detail [4,7,9]. This made it difficult to compare the neuronal cells of the camel STN, which were classified according to several somatic and dendritic characteristics, with their counterparts in other species, which were primarily classified according to the size and/or shape of the soma.

In this study, the presence and distribution of spines and appendages were used in the classification of neurons because they are functionally important components of neurons. These spines and appendages represent synaptic sites that allow neurons to engage in a very complex synaptic network such as glomeruli [19]. The spiny side branches (stalks) of the neurons of the camel STN were similar to their counterparts in other species. However, they arise not only from dendrites as previously reported in other species [4,7,9] but also from neuronal somata (this study). The presence of somatic spiny stalks for the stalked cells in the camel STN make them even more complex than their counterparts in other species. This study has also demonstrated pyramidal neurons—both spiny and aspiny. These neurons are similar to their counterparts found in other species but with additional somatic spines that have not been reported before. In addition, this study found small round or oval neurons (both spiny and aspiny) which most probably correspond to the small islet neurons reported in other species [4,7,9]. Some of these small neurons have a large number of spines. The density of these spines is much higher than the spines reported for islet neurons in previous studies [4,7,9]. On the other hand, the multipolar neurons found in the camel STN are similar to their counterparts reported in previous studies (5, 9). In addition to the aforementioned neurons, which are generally similar to their counterparts in other species, this study demonstrated distinctive types of neurons in the camel STN with unique features for some of them such as dendritic dilatations. 

The somatic spines, appendages, and side branches found for many neuronal types in the STN of camels in this study have not been reported in previous studies of the STN in other species [1,4,5,6,7,8,9,10,11,21,22]. Furthermore, at the electron microscopy level [24,25], the major component of the synaptic population in the CNS was formed mainly by buttons which synapse on spines and appendages. The presence of large numbers of spines and appendages of different forms for somata and dendrites indicates very complex synaptic activities and information processing in the STN. This suggests that the STN is a major site for the processing of painful stimuli and not just a relay site for these stimuli from the face area.

Some previous reports have described and stressed the importance of islet and stalked neurons almost to the exclusion of all other neuronal types in the STN [4,26]. In this study, although the islet and stalked neurons were the most commonly impregnated neurons, other types of neurons were also impregnated in large numbers. For example, this study reported large neurons with a soma diameter of up to 60 µm in some instances. Such large neurons have not been reported in the STN of other species. Moreover, neurons of unique shapes, such as octopus-like, boat-like, and lobulated cells, were exclusively reported by this study for the camel STN, while no similar neurons have been described previously in the STN of any other species. Notwithstanding, it is important to acknowledge the factors that affect the impregnation of neurons by Golgi methods, such as the selectivity of the Golgi method, the different Golgi methods used in different laboratories, the age of the animals, and the method of fixation, among others [27]. It is most probable that at least some of the neuronal types reported for the first time in this study might also be present in the STN of other species, but for unknown reasons, these were not stained.

Interestingly, this study reported unique feature of STN neurons for the first time, namely dendritic dilatations. Some neurons in the STN of the camels displayed these dendritic dilatations. In some cases, the dendritic dilatations were as large as the neuronal cell body. It is noteworthy that these dendritic dilatations were found in neurons of different sizes and shapes. These dendritic dilatations represent a unique neuronal morphological feature. To the best of our knowledge, no neuronal cell type in any other part of the nervous system of either camels or any other species has displayed such similar dilatations. The fact that no such dilatations have been reported in any other area of the nervous system may suggest that these dilatations represent a unique feature of the camel STN. Physiological studies might be useful to determine the significance of these dendritic dilatations in the camel STN. However, it is well known that the thicker the dendrites, the lower the electrical resistance they have [28,29]. Therefore, these dilatations may represent areas of low electrical resistance along the dendrites of different types of neurons in the camel STN. This feature, along with the findings that most of the neurons in the camel STN have many more appendages, not only on their dendrites, as the case in other species, but also at their somata, clearly suggests that the processing of pain and temperature in the camel STN is much more sophisticated compared to other species. If this proves to be true in future physiological studies, this will indicate that these special features of camel STN neurons represent developmental modifications to accommodate the sensory processing of the extreme temperatures and painful stimuli related to the camel’s habitat.

## 5. Conclusions

The neuronal morphological types described in this study represent the basis for our next investigations with the aim of characterizing these neurons in functional terms (e.g., connections, transmitters, and neuropeptides). This is part of an extended project aimed at creating a database of the neuronal cell types in various regions of the camel brain. In this study, we demonstrated different morphological neuronal cell types in the camel STN. The classification of the neurons in this study was based not only on the size/shape of the cell bodies but also the presence/absence of dendrite appendages and spines. Two types of unique neurons, namely the octopus and dilated types have not been described before. We speculate that these types of neurons have evolved as part of the adaptation of the camel’s sensory pathway to painful stimuli. Comparative neuroanatomical studies are important to improve our understanding of central nervous system organization, especially in a species such as camels living in rough environments in the desert.

## Data Availability

The data that support the findings of this study are available from the corresponding author upon reasonable request.
